# More Than a COVID-19 Response: Sustaining Mutual Aid Groups During and Beyond the Pandemic

**DOI:** 10.3389/fpsyg.2021.716202

**Published:** 2021-10-20

**Authors:** Maria Fernandes-Jesus, Guanlan Mao, Evangelos Ntontis, Chris Cocking, Michael McTague, Anna Schwarz, Joanna Semlyen, John Drury

**Affiliations:** ^1^School of Psychology, University of Sussex, Brighton, United Kingdom; ^2^School of Education, Language and Psychology, York St John University, York, United Kingdom; ^3^School of Psychology and Counselling, The Open University, Milton Keynes, United Kingdom; ^4^School of Humanities and Applied Social Sciences, University of Brighton, Brighton, United Kingdom; ^5^Overton Emergency Group, Lancaster, United Kingdom; ^6^The World Food Project, Hot Food for Hollingdean, Brighton, United Kingdom; ^7^Norwich Medical School, Faculty of Medicine and Health, University of East Anglia, Norwich, United Kingdom; ^8^NR2 Mutual Aid/COVID-19 Community Response, Norwich, United Kingdom

**Keywords:** mutual aid, COVID-19, SARS-CoV-2, community solidarity, community support, volunteering, social identity, pandemic

## Abstract

Mutual aid groups have been an indispensable part of the public response to the COVID-19 pandemic. They have provided many forms of support, in particular grocery shopping which has enabled people to self-isolate if required. While community solidarity during emergencies and disasters is common, previous studies have shown that such solidarity behaviors tend to decline over time, even when needs remain high. In this study, we address how mutual aid groups can be sustained over time in the context of the COVID-19 pandemic. We conducted 32 interviews with organizers of COVID-19 mutual aid and community support groups in the United Kingdom between September 2020 and January 2021. Based on a reflexive thematic analysis, we identified several community and group level experiences and strategies that were related to sustained participation in COVID-19 mutual aid groups. Meeting community needs over time with localized action and resources and building trust and community-based alliances were foundational elements in the COVID-19 mutual aid groups. Group processes strategies, such as a culture of care and support and regular group meetings, were used to help to sustain involvement. Some experiences resulting from participation in COVID-19 mutual aid groups were also related to sustained participation, including positive emotions (e.g., joy, pride), well-being and sense of efficacy, and an increasing sense of local community belonging and cohesion. Based on these findings, we propose four practical recommendations for sustaining mutual aid groups to assist public engagement with protective behaviors in the COVID-19 pandemic and beyond. We recommend providing practical and financial support to COVID-19 mutual aid groups; to mobilize the knowledge and the experiences acquired by COVID-19 mutual aid groups for developing programs and interventions for addressing the medium and long-term impacts of COVID-19; to prioritize community-level interventions; and to recognize the role of group processes as these have the potential to lead to long-term community responses. These approaches will be key for ensuring that communities effectively recover from the COVID-19 pandemic.

## Introduction

Community engagement is vital in strategies to combat disease outbreaks (Laverack and Manoncourt, 2015; Costello, 2020; Gilmore et al., 2020). This has been the case in the COVID-19 pandemic where in many countries mutual aid groups and other community support groups have been crucial in enabling self-isolation and shielding, in sharing information, and in encouraging vaccine take up (Pleyers, 2020; Sitrin and Sembrar, 2020; Tiratelli and Kaye, 2020; Costello, 2021; Mao et al., 2021). Community solidarity is common after disasters (Kaniasty and Norris, 1993, 1999, 2009; Beverlein and Sikkink, 2005; Drury et al., 2016; Ntontis et al., 2020), but these “disaster communities” typically decline over time as participants run out of energy and resources (Norris and Kaniasty, 1996; Kaniasty and Norris, 2004; Kaniasty et al., 2019). At the time of writing, the COVID-19 pandemic has lasted over 18 months, which means that there has been a prolonged need for community support and solidarity. Understanding “what works” in sustaining mutual aid and community solidarity groups is likely to have practical benefits in supporting public adherence to non-pharmaceutical interventions that are demanding (such as self-isolation) and will be useful for future crises. Yet, while there has now been some research on what COVID-19 mutual aid groups do (Mao et al., 2021) and on the predictors of participation (e.g., Mak et al., 2020; Wakefield et al., 2021), there is a lack of research on the experiences and strategies that help sustain these groups over time. Therefore, in this paper, we describe a study in which we interview organizers of mutual aid and other COVID-19 community support groups across the United Kingdom to address two research questions. First, what are the strategies employed by COVID-19 mutual aid groups to keep participants involved over time? Second, what are the experiences of participation and the consequences of involvement in the groups that have served to sustain participation? By addressing the question of sustained participation from the perspective of community organizers, this study aimed to contribute to a better understanding of the micro-processes involved in mutual aid groups during and beyond pandemic situations to help these groups endure in the long term.

## Community Solidarity in the COVID-19 Pandemic

In March 2020, increasing reported cases of a novel coronavirus (COVID-19) led the United Kingdom to implement several protective measures to contain community contamination, including a national “lockdown” (Public Health England, 2021). The population was asked to “stay at home,” only leaving the house for exercise once a day, for medical and food supplies, or for work if it was not possible to work from home. Millions of employees were put on the furlough scheme, a government support scheme in the United Kingdom that provided employers with the option to keep employees on the payroll without them having to work during the pandemic. People over 70 and those who were clinically extremely vulnerable were advised to shield, i.e., to stay at home, for 12 weeks. In addition, throughout the pandemic, anyone with symptoms, with a positive test, or in contact with someone with a positive test was required to self-isolate at home for 10 days or more.

Self-isolation can be extremely difficult and requires proper practical and financial support (SPI-B-Scientific Advisory Group for Emergencies, 2020; Patel et al., 2021; Reicher et al., 2021). Levels of adherence to self-isolation for the full period required tend to be low relative to other protective behaviors (such as physical distancing and mask-wearing), with financial constraints being one of the main reasons for failure to self-isolate (e.g., people who cannot afford to stop working) (Smith et al., 2021). The United Kingdom government offers financial compensation of £500, but this is less than the minimum wage and only about one in eight of the workforces are eligible (Reicher et al., 2021). The most comprehensive study of self-isolation in the United Kingdom (data from 53,880 people across 37 representative survey waves) found that shopping for food and other groceries was one of the main reasons people gave for breaking self-isolation (Smith et al., 2021). Support for self-isolation has been considered particularly critical among ethnic minority groups and/or low-income and vulnerable populations (Hooper et al., 2020; Kerkhoff et al., 2020), with evidence showing that the access to social support increases adherence to self-isolation measures (Kerkhoff et al., 2020).

While in some countries the state offered wrap-around support (Patel et al., 2021), in other countries, most of the practical support to help people self-isolate (including shopping and collection of medicine) has been provided by members of the community who self-organized in groups to help their neighbors (Al-Mandhari et al., 2020; Pleyers, 2020; Sitrin and Sembrar, 2020). In the United Kingdom, in the early days of the pandemic, more than 4,000 mutual aid groups were created across the country (Booth, 2020). Additionally, many new community support groups sprang up that did not call themselves “mutual aid,” and many existing community organizations changed their focus to provide COVID-19 support. In this paper, we use the term “COVID-19 mutual aid groups” to refer to all of these groups, acknowledging this diversity and including both emergent and pre-existing community support groups. COVID-19 mutual aid groups also varied in their level of politicization and understanding of mutual aid (Firth, 2020; Mao et al., 2021). Some groups but not others consciously drew on the mutual aid tradition. The term “mutual aid” was first introduced by Kropotkin (1902), a well-known anarchist thinker. Practices of mutual aid are widely present amongst social movements and anarchist settings (Firth, 2020; Spade, 2020). In these contexts, the slogan “solidarity not charity” is used to erase distinctions between helpers and helped to prefigure social change (Firth, 2020). Mutual aid groups are spaces that cultivate solidarity amongst people that have come together to address a shared need or concern (Spade, 2020).

Mutual aid groups helped to create hope in times of coronavirus (Mahanty and Phillipps, 2020) and the support they provided was an essential part of the public response to the COVID-19 pandemic in the United Kingdom (Tiratelli and Kaye, 2020). These groups focused on building bottom-up structures of cooperation and horizontal networks of solidarity (Whitley, 2020), which represents a radical divergence from traditional public services and forms of volunteerism (Spade, 2020). Many different acts of solidarity during the COVID-19 crisis have been reported, including grocery shopping and delivery, food parcel deliveries, collection of prescriptions, dog walking, postcard and library services, emotional support by telephone/email helpline, informational support on existing public services, community gardening, and more (O’Dwyer et al., 2020; Tiratelli and Kaye, 2020; Mao et al., 2021, in press). Most of the published research on COVID-19 mutual aid groups has been descriptive (see Mao et al., 2021 for a review). However, some recent work has started to examine psychological processes (Bowe et al., 2021; Wakefield et al., 2021; Mao et al., in press), which is useful for addressing our questions of how such groups can be sustained.

### The Dynamics of Solidarity in Extreme Events

An extensive body of research evidence has shown that cooperative and solidaristic behaviors – meaning forms of support provided for others because they are fellow members of one’s community or group – are common among those affected in the immediate aftermath of disasters and extreme events (Beverlein and Sikkink, 2005; Drury et al., 2016; Ntontis et al., 2020). Two types of explanations have been offered for solidarity behaviors among those affected by disasters: social capital (in which people draw upon existing connections with others) and emergent groups (in which connections are created in and through the disaster). In each case, shared social identity – seeing others involved as an “us” or “we” – motivates and enables cooperation and support and allows people to act as one (Drury et al., 2019).

In the COVID-19 pandemic, initial research looked at the emergence of mutual aid through a social capital lens (Felici, 2020), in which solidarity in affected communities is explained based on existing social networks, trust and reciprocity (Jovita et al., 2019). Indeed, a rapid review of the literature available up to October 2020 showed that social networks and connections, local knowledge and social trust were key dimensions associated with COVID-19 community organizing and volunteering (Mao et al., 2021). Further recent studies have examined social psychological processes in COVID-19 community solidarity groups, by looking for instance at participants’ representations of citizenship (O’Dwyer et al., 2020), and the role of community identity as a predictor of providing COVID-19 help among volunteers (Wakefield et al., 2021). Cocking et al. (in preparation) concluded that mutual aid groups were based on a mixture of social capital and new emergent groups that evolved in response to participants’ desire to create new forms of identification with one’s own neighborhood or street. These findings suggest the relevance of looking at mutual aid groups from a social identity perspective, that considers the role of identity dynamics in understanding COVID-19 support (Stevenson et al., 2021).

Moreover, although there are some examples of long-term community solidarity in recovery processes (see for example Occupy Sandy, Bondesson, 2020), there is a tendency for a decline in community support in the recovery and rebuilding phases of disasters (e.g., Kaniasty and Norris, 1993, 1999, 2009; Ntontis et al., 2020). Among other aspects, this deterioration path has been explained in terms of the disruption of social networks after the disaster (e.g., death, relocation), a decline in terms of resources available (which decreases expectations of support), and the possibility of experiencing long term stress that may lead to fatigue and saturation of support networks (Norris and Kaniasty, 1996; Kaniasty and Norris, 2004). In Ntontis et al.’s (2020) study of people 15 months after they were affected by a flood, those no longer participating in the solidarity group described how the social identity associated with the flood had become less important to them over time, compared to their other identities. But while the common fate that brought people together declined as the memory of the flood receded, many were still struggling with secondary stressors such as rebuilding their homes and in need of support (Ntontis et al., 2020). Considering the ongoing and expected long term-social impacts of COVID-19 (Bedford et al., 2020), particularly for vulnerable groups and communities and those who still need to self-isolate, understanding the psychological processes that can help sustain community solidarity post-COVID is critical (Al-Mandhari et al., 2020).

### Understanding Sustained Participation in COVID-19 Mutual Aid Groups

Only a small number of existing studies have examined the strategies used by groups to sustain solidarity after a disaster. These studies provide suggestive evidence that the following strategies may be important to sustain emergent mutual aid groups: invoking the group identity in discussions; commemorations and other public events; support from allies; and group meetings (Ntontis et al., 2020; Tekin and Drury, 2020).

Research on collective action and psychological effects of participation in volunteering provide some further suggestions on factors that could be important in sustaining solidarity over time. Although collective action and volunteering are usually addressed from two different theoretical perspectives, COVID-19 mutual aid groups seem to have elements of both. COVID-19 mutual aid groups created opportunities for people to act collectively by working together to achieve a common objective (e.g., to support the local community) and simultaneously facilitated volunteering among community members.

The literature on collective action suggests that perceptions of success and efficacy can motivate continued involvement (Van Zomeren et al., 2012; Becker and Tausch, 2015; Saab et al., 2016). Collective efficacy in the context of collective action often encompasses not merely the feeling that something can be done, but that one’s own group can do it (Gamson, 2013). Recent experimental studies have shown that collective efficacy affected collective action intentions only when hope was high (Cohen-Chen and Van Zomeren, 2018). Previous field studies have been also showing that positive emotions, such as hope, play a crucial role in mobilizing individuals to take part in collective action (e.g., Wlodarczyk et al., 2017).

Furthermore, Vestergren et al. (2018), based on interviews with environmental campaigners, found that participation in collective action led to several sustained psychological changes such as feelings of empowerment, self-confidence, personal relationships, and changes in consumer behavior. In addition, the study showed that stronger and continued relationships among the campaigners facilitated these psychological changes over time (Vestergren et al., 2018). Other literature has found that sustained collective action is also influenced by interpersonal relationships and organizational mechanisms, suggesting the need for developing collective coping strategies, collaborative relationships, and to allow some flexibility in terms of roles and procedures within the organization (Mannarini and Fedi, 2012). Recent literature on activism burnout similarly proposes a community-care burnout orientation, which suggests looking at burnout as a part of activism and as influenced by the organizational context, rather than as something that individual activists experience outside of activism (Gorski, 2019).

Recent studies also suggest that positive emotions arising from the experiences of participation in COVID-19 mutual aid groups may be particularly important for understanding sustaining participation. In this regard, a interview study with people involved in COVID-19 mutual aid in the United Kingdom showed that the experience of participation may have affected participants’ wellbeing through positive emotional experiences, improved social relationships, increasing sense of purpose in life, and greater sense of control (Mao et al., in press).

The literature on volunteering supports the need to look at the positive outcomes of participation, with a meta-analysis showing that volunteering has favorable effects on well-being, life satisfaction and depression (Jenkinson et al., 2013). Additionally, increasing social ties is often a benefit not only for long-term volunteers, but also for short-term and/or occasional volunteers (Hyde et al., 2014). Similar outcomes have been found in the COVID-19 context, with a recent study showing that community helping predicts community identification and unity during the pandemic, which in turn seems to increase well-being (Bowe et al., 2021).

## The Present Study

In this study, we interviewed coordinators or organizers of COVID-19 mutual aid groups in different areas of the United Kingdom. First, we aimed to examine any strategies employed by the groups for sustaining participation among volunteers. Secondly, we aimed to examine the experiences of participation and outcomes of involvement in the groups that have helped to sustain participation. We followed a qualitative approach, as this is particularly useful for exploring people’s experiences in a flexible way, particularly when there is a need to be open to unexpected findings (Rogers and Willig, 2017). We chose to interview only organizers as these are the people in groups who would consciously take decisions and actions to recruit volunteers and attempt to encourage sustained participation, and so would be able to provide insights on strategies used. We also expected that they would also be able to describe experiences in COVID-19 mutual aid groups that might be important to explain how participation can be sustained over time. Based on previous findings (Ntontis et al., 2020; Tekin and Drury, 2020), we expected that strategies such as facilitating and invoking a sense of identification, group events, collaborations and alliances, and group meetings would help to sustain COVID-19 mutual aid groups. In addition, we anticipated that positive emotions related to participation, feelings of efficacy, empowerment and sense of belonging would be considered critical in sustaining participation (Cohen-Chen and Van Zomeren, 2018; Vestergren et al., 2018; Mao et al., in press).

## Materials and Methods

### Participants and Recruitment

Thirty-two semi-structured interviews were conducted between 10 September 2020 and 6 January 2021. Participants were approximately between 20 and 75 years old, 17 were female and 15 male. The average length of interviews was 60 min. All were organizers rather than simply volunteers in mutual aid groups. Twenty-four interviewees were coordinators of mutual aid or community support groups in England, four in Wales, three in Scotland, and one in North Ireland. Participants were recruited through multiple channels. A call for participants was disseminated through diverse networks (e.g., the Communities Prepared program) and social media accounts (e.g., professional and personal Twitter accounts). The call for participants stated that we wanted to interview organizers of COVID-19 mutual aid or community support groups. We also directly contacted mutual aid groups across the country with an invitation to participate in the study. These groups had their email addresses publicly available and were identified through searches on Facebook and national networks of mutual aid groups (e.g., COVID-19 Mutual Aid United Kingdom). We approached both pre-existing and emergent groups and we sought variability in terms of geographic location, areas of intervention (e.g., shopping groceries; helpline support), and socio-demographic characteristics of the participants.

Mutual aid groups were invited to share their experiences and views on the factors that enable mutual aid groups to endure. If groups showed interest in participating, more details about the study were provided in a detailed participant information sheet. We interviewed one participant per group, except for three groups for which two organizers were interviewed from each. Therefore, we spoke to organizers from 29 different groups. A few interviewees also mentioned being involved in more than one group (including in pre-existing and emergent groups), although they ended up focusing the interview on the group they were more strongly engaged with. Potential participants were asked to give written informed consent. We offered a £20 voucher as compensation for participants’ time and interviews were conducted on Microsoft Teams and Zoom.

### Interviews Schedule and Procedure

Interviews covered questions on the mutual aid group’s story, the participant’s own experience, activities carried out by the groups, issues related to organizing, motivations for participation, changes and problems in the group, strategies that have helped keep the group going, and lessons from coordinating the group. See the full interview schedule in the supplementary material.

Twenty-eight interviews were conducted by the first author and four by the second author. Following the first four interviews (conducted in September 2020), slight modifications were made to the schedule. We added introductory sentences before each block of questions (e.g., “now, I want to ask you some questions about your role in the group”) and two new questions (“have you had any previous experience of organizing groups like this?”; “how do you see the future of this group?”). The revised schedule was re-submitted for ethical approval, which was obtained around mid-October. Most of the interviews were then conducted between the end of October and early December. We planned to end data collection in mid-December 2020, but one of the groups who accepted to participate in our study asked to postpone the interview. Thus, the last interview was conducted on January 6, 2021.

The interviews were transcribed verbatim by a single professional transcriber. All personal data collected was kept strictly confidential in accordance with the Data Protection Act 2018. Any reference to individuals or specific locations were anonymized and participants’ names were replaced by pseudonyms.

### Analytic Procedure

We followed a reflexive approach to our Thematic Analysis (TA) (Braun and Clarke, 2019). The first author led the analysis process, but all stages were discussed with co-authors who also engaged with the material. We used the NVivo software to assist the organization of the codes and the initial themes. The data set was analyzed without a pre-existing coding frame but informed by our research questions and theoretical assumptions. Specifically, we were interested in group processes (and other psychological factors) involved in sustaining participation in COVID-19 mutual aid groups, and in particular the strategies that were employed by organizers and the experiences of participation that were related to sustained participation. Thus, our approach was theoretically driven, yet our interview questions and analytic approach also allowed for the identification of other psychological factors than those expected.

Although presented as linear (see Table 1), the analysis process was dynamic, iterative, and involved continuous reflection and discussion as suggested for reflective TA (Braun and Clarke, 2006, 2019). Three of the co-authors were active members and organizers of COVID-19 mutual aid groups, and their insights were particularly useful for data interpretation in later phases of the analysis.

**TABLE 1:** Six-phases thematic analysis.

Phase one – Familiarizing	In the first phase, we familiarized ourselves with the data set, by reading, re-reading, and taking notes. These notes were shared and discussed with three of the co-authors.
Phase two – Coding	The second phase consisted of the development of several codes (e.g., “wanted to help others”: “feeling part of something”). This stage followed a bottom–up approach, and the codification process was data-led.
Phase three – Initial themes generation	The third phase involved an initial generation of themes. We identified links between different codes, and several codes were re-organized in initial themes. We developed six major superordinate themes involving aspects related to group emergence and development; coordination, organization and cooperation; individuals in the group, from roles to motivations; group changes over time, strategies for keeping the group going, and consequences of participation. In this phase, several subthemes were also created. For example, the initial theme focusing on motivations for participation was organized into several subthemes (e.g., sense of responsibility and community; coping with lockdown).
Phase four – Reviewing themes	In this phase, we reviewed all the themes and subthemes. All extracted quotes were re-analyzed, and several merging and splitting were made. During this reviewing process, we focused on the themes and subthemes that were mainly related to our overarching research questions on sustaining participation in mutual aid groups.
Phase five – Naming the final themes	The fifth phase involved defining and naming the final themes. Five interlinked themes were generated: meeting community needs over time with localized action and resources; building trust and community-based alliances; employing group processes strategies; experiencing enjoyment and efficacy in collective coping; an increasing sense of local community belonging and cohesion.
Phase six – Writing the analysis	This phase involved writing the analysis, highlighting the interlinked nature of the generated themes. To support our interpretations, we use several interviewees’ quotes.

The themes presented in this paper show patterns of shared meaning that apply to the entire data set (Braun and Clarke, 2006, 2019). Overall, the generated themes allow us to identify and discuss interviewees’ understandings of the strategies perceived as key for sustaining mutual aid groups over time, and the role of different types of experiences of participation to sustained community solidarity.

## Results

Most of the groups in this study were created at the beginning of the first United Kingdom national “lockdown,” in March 2020. As in other countries (Pleyers, 2020; Sitrin and Sembrar, 2020), people from existing activist or volunteering circles played an important role in some groups; and in some communities previous and current local authority councilors took a lead. Other groups were created by people without any previous experience of participation. In fact, while several participants mentioned previous experience of volunteering, activism or community organizing, nine participants did not have any previous experience of participation before the pandemic. Despite these differences in background, our participants shared a common orientation toward local community needs and were all involved in organizing and providing multiple forms of COVID related support. They offered many activities and services, including practical support (e.g., grocery shopping, collecting prescriptions), information support in different languages (e.g., development and distribution of pamphlets), emotional support (e.g., telephone/email helpline), and financial support (e.g., solidarity funds, foodbanks). Ultimately, groups organized help based on what was perceived as necessary in their local community at a given time. Pre-existing groups reorganized their activities and services to respond to current needs. Emergent groups, in turn, organized around needs perceived as not being addressed by charities, existing community groups, or local authority services. Based on the accounts of organizers, the five generated themes presented below (Table 2) report the most prevalent strategies or experiences sustaining COVID-19 mutual aid activity. Themes 1 and 2 address some of the foundational elements in COVID-19 mutual aid groups. Theme 3 is essentially about the group processes strategies that help to sustain involvement within the group. Themes 4 and 5 are about the experiences resulting from such involvement that helped to bring about efficacy, positive emotions, and sense of belonging.

**TABLE 2:** Overview of the generated themes.

**Name**	**Description**
Theme 1: Meeting community needs over time with localized action and resources	The first generated theme focuses on the localized dimension of practical and human resources that were mobilized by mutual aid and community support groups. It includes details on how groups organized locally around community needs and how the mobilization of local resources was a key aspect for sustaining the group.
Theme 2: Building trust and community-based alliances	This theme includes references relating to the strategic alliances between groups, institutions and charities. Participants’ views on the need and importance of building trust and alliances within the local community are represented in this theme.
Theme 3: Employing group processes strategies	The focus in this theme is on the intentional and conscious things done by organizers to sustain the groups. These strategies involve invoking identification, group care, facilitating communication, an informal but organized leadership structure, and group’s meetings and events.
Theme 4: Experiencing enjoyment and efficacy in collective coping	This theme addresses the positive experiences and benefits for those who were involved in organized community solidarity during the COVID-19 pandemic.
Theme 5: Increasing sense of local community belonging and cohesion	The focus in this theme is on the impact of COVID-19 community solidarity for dimensions related with the community, namely sense of belonging and cohesion. These dimensions appear related to ideas of group continuity beyond the pandemic.

### Theme 1: Meeting Community Needs Over Time With Localized Action and Resources

COVID-19 mutual aid groups participating in our study emphasized a localized approach in their action and relied on donations and volunteers from within the community: “all the donations come from people, just people, we would just do these posts and then people would just come in and just give their unwanted stuff or they buy us, buy food” (Ivy, Greater London, England). Other participants described how pre-existing charities and food banks offered donations or made their resources (e.g., vans) available to recently formed groups: “we have access to a minibus as well through [charity organization name], so our transport issues have mainly been quite easy.” (Joshua, North East England). Existing community centers opened their kitchens so newly created groups could cook and distribute hot meals to those who needed them: “we just use the venue, we used the community center because no one else was using it, so we just, like, spilled everything out, all these different food providers just had stuff all over the community center” (Evelyn, South East England).

The relationship with local businesses and companies was also mentioned, as they had donated food and other grocery items: “well we’ve got support from the local businesses” (Rose, Mid Wales). Local printers for example donated leaflets and posters that were then distributed by local volunteers: “so, we produced this, we got, we got funding, well we got sponsorship from a local printer” (Matthew, West Midlands, England). Additionally, volunteers used their own vehicles, laptops, phones and other personal resources: “is just volunteers, just do it. You know, everybody pays their own petrol to go to the foodbank, or, you know, whatever.” (Karen, South East England).

Some groups were also able to receive funding from the city or/and town councils, churches, or local rotary clubs: “well the town council gave us grants”; the district council have supported us; both our rotary clubs have supported us. The churches together in (city) have supported us (Laura, South East England). A few participants mentioned that they had received grants from national foundations (e.g., Scotland Foundation) or other national lottery funds, but most groups relied only on local and small grants: “a couple of funds yeah (…)^1^. And then we’ve had one or two other funds in, I think through the community foundation. And some other small grants.” (Luke, South Wales).

Despite the recognition that *access to local resources* such as funds, vans, and venues was important, many participants argued that the most valuable resource was the volunteers: “Us! We have us” (Karen, South East England). All participants stressed that providing community support was possible because many people were willing to help, had the time and the resources to actively engage in mutual aid groups or to donate goods or money to support fundraising events:

Interviewer: What resources did you have?
Aurora: None.
Interviewer: None, okay.
Aurora: I mean, well, it depends on what you mean by resources, if you mean funding and finance no. But we do have a lot of social capital in this area. So, there were a lot of kind of willing and capable people that wanted to help their community and people who were happy to use their own resources in terms of like printing out leaflets and delivering them around and that kind of stuff, donating food to the food bank, that kind of thing. So, I think if we’d have lived in a different area it wouldn’t have been possible without some external funding. (Aurora, East of England).

Aurora’s comment suggests the role of pre-existing social capital in explaining support and help in her community, which aligns with early analysis of the emergence of COVID-19 mutual aid groups in the United Kingdom (Felici, 2020). However, she also pointed to the importance of external funding for groups working in more deprived areas. In fact, participants from groups working with marginalized groups and/or in deprived areas mentioned the need for fundraising and were more concerned with access to funding. When discussing existing resources such as funding and access to venues, a few participants also stressed that the COVID-19 lockdowns created an exceptional time where some resources were available free of charge: “venues are going to be back in use, and we’ve been using a venue for free, and that will change. I don’t suppose that’s sustainable for the venue, and the funding’s going too.” (Evelyn, South East England).

As with Evelyn’s comment, other participants mentioned losing access to venues and storage spaces and recognized that accessing to funding and resources would be more complicated after the pandemic, even if the need to provide support will continue to exist. Interviewees also expressed concern over a decline in the number of people with time and resources to volunteer due to the end of furlough. Indeed, several participants mentioned that their group has already started to lose volunteers: “so, we lost a lot of people after lockdown when the government said that [people] had to go back to work” (Sophie, East of England).

Interviewees emphasized the *profile of the volunteers* involved in COVID-19 mutual support as a factor in sustaining the group, as “really skilled people” (Arthur, South West England) who had time to volunteer in the community, in some cases for the first time in their lives: “You know, we had nurses and web designers and – people just come out of the woodwork. Especially when they’re on furlough and can’t work. I think it would have been very different if people were having to work.” (Denis, South East England). Other participants mentioned the key role of specific experiences and skills such as: organizational and teamwork; experience in applying for funding; experience in public health and social services (e.g., ex-social workers); experience in community organizing and project management; IT and digital skills; leadership and communication skills. In general, participants mentioned several of these skills simultaneously and considered them as key factors in explaining how the group responded to the pandemic. Importantly, most participants describe their communities as having the necessary skills and resources to provide community support during the pandemic. Furthermore, the local level of action also facilitated engagement in mutual aid and community support during the pandemic:

Interviewer: Okay, so finally, I want to ask you if you learned something from coordinating this group?
Lisa: I think what I have learnt is that it is easier to ask or to invite people to volunteer in their own neighborhood where they live. (…). Because it’s time limited, they know that it’s how long it’s going to take them to get there, they know how long it’s going to take them to get back they know (…). Local volunteering initiatives are easier to keep going and to operate than something which stretches over a wider field and where traveling is required. (Lisa, South East England).

As with Lisa, other participants mentioned that acting locally involved less time and effort, which may have reduced barriers for participation and acted as an encouragement for participation during the pandemic.

Thus, our analysis suggests that COVID-19 groups participating in our study followed a localized approach, which was perceived as the best level of action to respond to communities’ needs. The local dimension of resources, including the human resources (i.e., volunteers), had an essential role in mobilizing and sustaining COVID-19 mutual aid groups over time. Specifically, it facilitated the mobilization of resources, as well as the coordination and distribution of help within local communities.

### Theme 2: Building Trust and Community-Based Alliances

Twenty-three interviewees mentioned that *building alliances* within the community was a key aspect facilitating the organization and the provision of help. These collaborations involved other mutual aid groups, foodbanks, community centers, youth groups, charities, as well as local pharmacies, local public bodies, among others. The cooperation involved sharing resources and knowledge:

*We work very closely with other groups. We’ve actually started workshops for our coordinators with other organizations, so we have a local organization (…), so they did a workshop with us so that coordinators, so that we can pass referrals to each other. A lot of the people that come through to us may not have heard of the other organizations who can offer support. So that when we’re building relationships with the person that we’re supporting, if they have additional needs, we can then refer back to other organizations. (Lucy, West Central, Scotland).*

As with Lucy, many other participants mentioned *cooperation between organizations and groups* as key for organizing and distributing services within the community and to ensure that all people, streets and neighborhoods were covered: “the idea to manage the community groups in the area wasn’t to overtake them like other areas have, it was actually to work with them” (Joshua, North East England). In some cases, the collaborative approach between groups and organizations facilitated access to venues and storage spaces. In other groups, access to funds by new and emergent mutual aid groups was only possible through pre-existing registered groups who had formal recognition: “we were able to use the financing team of the housing association to manage the funds, disperse the funds” (Logan, West Central, Scotland). Indeed, some groups applied for funds through pre-existing community interest organizations, who acted as intermediaries between funds agencies and informal mutual aid groups.

Moreover, *alliances with pre-existing organizations* also facilitated the relationship with marginalized groups and communities. Ivy, whose group was working with migrant and refugee people, explained:

*The way that we reached the people [migrants’ communities]^2^ that we were trying to work with was through these well-established projects and charities (…), and organizations, these long-established projects and rightly so they were very, very protective, because of the real kind of sensitivity around these people’s situations. So, it was really difficult because really you needed to be, we needed to have, they needed to foster a sense of trust of who we are and what our intentions were before they were ready to hand over you know their clients. So, it took a long time. (Ivy, Greater London, England).*

The importance of *building trust* between the group and the community was expressed by 13 participants who agreed that trust has been one of the most important aspects in the endurance of mutual aid groups over time: “I guess the main thing is just trust and relationship with the community” (Logan, West Central, Scotland). The emphasis in building trust was particularly evident in participants whose groups were working directly with migrants, refugees, and Roma people:

Interviewer: You mentioned some reasons connected with [the term] mutual aid, which ones were important for your group?
Theo: We, from the very beginning, we were very clear that we were a community organization; we’re not a local government led organization. (…). We don’t share it [people’s information] with anybody, we don’t act as border police, we’re not here to judge people’s needs, or make assumptions about their backgrounds, or why they are or why they, they need help. I think if we had gone down the line of registering ourselves as a company and doing it in that sort of charity or whatever and doing it in that sort of official way, there are loads of things that we would not have been able to do. And also, with a lot of communities, especially migrants and refugees, you lose the trust as well. (Theo, Greater London, England).

By stressing the importance of trust when working with marginalized communities, this participant also expresses a fundamental difference between charity and solidarity. Overall, COVID-19 mutual aid groups offered help without defining any criteria of eligibility. Any person in the local community could receive support if they asked, and this was perceived as particularly important to reach people in need that may have been considered not eligible for receiving social benefits or support from charities in the past.

The relationship with the local authority was also addressed during the interviews, with participants sharing mixed experiences. A group of participants mentioned disappointment with the lack of response from their local councils: “the council weren’t giving us funding, they weren’t, you know, opening up their buildings, they weren’t really doing anything that could support the efforts. So, it’s, they were a bit disappointing, a bit disappointing.” (Logan, West Central, Scotland). The excess of formality and rules, and the lack of practical help and support provided to mutual aid groups was expressed by this set of participants:

*There was no, none of them offered any help either, whether it be practical, yeah, they gave some money and donation which was lovely, but it wasn’t really that that we wanted. You wanted actually them to get involved and none of them ever did that. Neither the parish councils nor the borough council ever did that. (Sophie, East of England).*

Other participants mentioned support from local government and described the relationship as positive and helpful. For these participants a positive relationship facilitated access to specific resources and knowledge: “so, we use the parish council website, all this information is up on their website” (Jack, South East England). Most participants who mentioned positive experiences with their local councils also mentioned some previous experience of participation as councilors or involvement in pre-existing organizations:

Interviewer: Okay, and how is the, the relationship, well the relationship with the local council?
Freddie: The local council? Very good I would say. So, so we’ve got, with the, with the community council. I’m going to say that because I’m the community, one of the community councilors anyway. And, in the other, the other group I run now with two other community councilors and the local authority councilor as well. (…) So that, that helps in that sense. (Freddie, South West, Wales).

Overall, participants expressed the importance of building trust between the mutual aid group and the community, and in particular trust with established organizations and bodies; other support groups; and the sections of the community most in need. Our analysis also suggests that alliances with other groups facilitated continuity of the group activity. In some cases, mutual aid groups shared knowledge (e.g., about existing services within the community), resources (e.g., donations), including human resources. Some participants mentioned being involved in other groups and community organizations, and that other volunteers in their group have also started to provide support to other local organizations (e.g., foodbanks). This suggests that community alliances facilitated sustained community solidarity through the group, but also beyond it.

### Theme 3: Employing Group Processes Strategies

COVID-19 mutual aid groups each comprised a small number of people organizing and coordinating local support and help. Groups were organized at the village, ward, neighborhood, or street levels. Our interviewees were, in some cases, part of a subgroup composed of coordinators, and for some participants there was a clear distinction between the coordinators and the volunteers. *Shared identification and sense of belonging* to the COVID-19 mutual aid group were explicitly mentioned by 17 participants as an important part of the experience and as a strategy for sustaining involvement:

Interviewer: And do you think that applies to your own group as well, I mean do you think your volunteers initially felt some sense of being part of a group?
Denis: Yeah, definitely, definitely, and a lot of people were very reflective about being part of the group, there were, there wasn’t just a lot of comments about how they would help people or say they would put things on there [WhatsApp, Facebook] about how happy, pleased they were about being part of a group of people who were doing this. Who were, were, such a nice group of people. There were a lot of reflective statements. (Denis, South East England).

As we can see from Denis’s comments, this sense of belonging to the group was perceived as valued by the members of the group and was positively described by several participants. Likewise, the sense of group was also promoted by some organizers: “although of course I try and make sure we all look after each other as a team. But I think working together as a team has been what overcomes different issues.” (Luke, South Wales).

Furthermore, engagement involved high commitment, with 22 participants explicitly mentioning an immense amount of work, particularly during the first national “lockdown” (between March and July 2020). In many cases, a structure for providing help was non-existent, and participants had to create everything themselves. While this worked as motivating factor for some participants who felt the need to set up the group and keep going, some participants explicitly mentioned feeling extremely tired: “to be honest I feel absolutely worn out which is probably why my health isn’t great. It’s hard.” (Olivia, South East England). Some groups were starting to implement measures to avoid personal burnout, such as delegating work:

*The second-best thing we ever did, was Noah got a deputy and so did I, because we were doing this for 7 days a week. Probably for the first 6 or 7 weeks, and it was hard, because it was 12-h days. (Sophie, East of England).*

However, there were not many references to strategies of personal care, although participants stressed many times the importance of avoiding burnout. Participants assumed the “exceptionality” involved in the times they were living in, and argued that once the organizational structure (e.g., procedures for task allocation) was implemented the amount of work became more manageable. There was also an active effort from organizers to make things easier for volunteers:

Interviewer: Has involvement in the group meant a lot of time and effort for you, for the others?
Noah: Yeah, I think, what we tried to do, is something I generally try to do, is make things very simple for people. So, we set up those processes and mechanisms to make sure that people had the least amount of work to do, so, when it came to somebody having to do a shopping task it was, “right, here’s the shopping list, here’s the phone number of the client, please phone them up and let them know when you are going to go, talk about any issues.” All they had to do was that, do the shopping, send us a receipt, job done basically. (Noah, East of England).

Moreover, the importance of *caring and supporting group members* was explicitly mentioned by 22 participants. Interviewees stated they had made strenuous efforts in preparing and elaborating clear guidelines to protect volunteers and avoid the risk of spreading the virus when providing support to others. Simultaneously, interviewees also mentioned the importance of ensuring that no volunteer would get overloaded, of distributing the workload fairly, and of providing emotional support to volunteers when necessary: “a lot of the time it’s just been giving people time and a listening ear, so I think that’s been really important for folks” (Logan, West Central Scotland). This kind of emotional support within the group was mentioned by several participants as a key aspect helping to maintain mutual aid group members active and motivated:

Interviewer: Okay, and how about the kind of things that have helped to keep the group going? Can you mention specific things that you have done that maybe helped the group?
Emma: I think support, support and teamwork [explains] a lot of it. I think doing this kind of thing, and especially doing it now and working from home, now you’re back in and stuff like that, you need a good team around you, and you need that support and that motivation and that encouragement (…). So, I think, because we’ve had that, it supported us, it supported the volunteers and gave them reassurance as well. (Emma, North West England).

As part of this culture of group care and support, 20 interviewees mentioned that they actively tried to keep the *communication regular within the group*, including by asking volunteers about their needs regularly, and trying to respond to these needs. To facilitate internal communication, participants used WhatsApp and Facebook groups, and regular telephone calls. Other groups organized regular meetings (e.g., weekly, fortnight, monthly). While most meetings were online, some groups had the opportunity to meet outdoors:

*For example, the helpline people who probably were the busiest of the volunteers, we [the organizers] would have a weekly get together with them, on Zoom, not physically. And let them share experiences, so they had a really high degree of camaraderie. We also had a group called the communications group which was, a cross-village group, that we put together to try to get over this lack of communication problem that we have in the village. And that sort of became more a cross-village advisory group. And I think that level of communication, bringing people together and just letting them share the good and the bad, helped keep people together. (Karen, South East, England).*

Karen’s excerpt introduces the importance of communication at different levels, within the group, but also within the community. Moreover, she also makes a clear distinction between the group’s coordinators and (“we would meet with them”) and the volunteers’ group. This approach was shared by other participants, who mentioned separate meetings with the group of coordinators and with the volunteers. The few participants who were involved in superordinate mutual aid groups in urban settings highlighted their effort to support street coordinators, and mentioned regular meetings with the coordinators, who were then responsible for liaising with the volunteers at the street and neighbor level:

Interviewer: Okay, and how about the things that maybe you have done as coordinator that have helped keep the group going? What kind of things did you do to sustain the group?
Lucy: I think the main thing I do to help sustain the group is to support the coordinators. The coordinators are the ones that are in direct contact with our volunteers. We hold fortnightly meetings with the coordinators so we’re able to, you know, talk about the various things that are happening or have happened within the group over the last 2 weeks. It’s all about communication. I think that’s why our volunteers, they feel part of the organization. We update them regularly on things that are happening within the organization. (Lucy, West Central, Scotland).

Lucy, like other interviewees, reinforced the importance of communication so volunteers feel part of the organization. She did that by highlighting the importance of supporting the coordinators who would in turn support the volunteers. Additionally, *getting together for socializing* was considered important for sustaining the group over time, and some groups were able to organize outdoor meetings or planned to do so as soon as possible. Other groups created socializing online spaces (e.g., WhatsApp group, Facebook group). Many participants recognized the importance of socializing moments and expressed their intention to organize events in the future, when the pandemic is over, so volunteers could all meet each other face-to-face, some for the first time, and celebrate their achievements.

Most groups involved in our study did not have legal status nor formal chairs, but there was, in most cases, a structure of coordination involved. *Shared leadership* by a small group of people was, in most cases, assumed informally and spontaneously. But it was considered an important aspect for sustaining engagement over time: “I noticed that the groups that do have that kind of central organizing. They’re much more coordinated in terms of reaching out for help and so on.” (Theo, Greater London, England). However, participants seemed to value sharing responsibilities in terms of decision making and the lack of formal rules (e.g., chair) involved in the idea of mutual aid. Ultimately, even in groups that had a clear distinction between coordinators and volunteers, participants claimed to have approached things as a group, and that there was a shared goal that helped to sustain group activity over time: “I think we worked well because it was about the community, it wasn’t about us, for most of us anyway.” (Rose, Mid Wales).

In summary, interviewees referred to many conscious and intentional ingroup strategies as important for sustaining participation in the groups participating in our study. Essentially these strategies revolved around promoting a shared identity, effective communication between group members, a culture of care and support within the group, group meetings and events, and an informal but organized leadership structure.

### Theme 4: Experiencing Enjoyment and Efficacy in Collective Coping

Most participants expressed positive emotions associated with their and others’ participation in mutual aid groups during the pandemic, such as joy, pride, and happiness:

*I think there’s a hard core of people who really enjoy just helping and supporting. For no other reason, you know, I enjoy what I do in my other activities, it means sometimes two hundred mile a day driving patients, but I know they’ve had their radiotherapy – it’s very rewarding. (Ryan, East Midlands, England).*

Like Ryan, other participants used expressions of *joy* to describe their and others’ experiences. Expressions such as “there was a lot of enjoyment” or “they [volunteers] were quite pleased” were used by several participants. Additionally, there was a strong sense of accomplishment and pride among some participants: “so, I think quite a lot of the members who were quite involved in it did feel quite proud” (Oscar, South East England). Importantly, participation was also considered a form of coping with lockdown measures and volunteering was considered “win–win” situation with benefits for volunteers and for the whole community:

Interviewer: You were saying, explaining how you started?
Amelia: Yeah, it was very important to get the message out that it wasn’t just for people in difficulty, it was for everybody, it was to help stop food wastage. It was, it was for everybody in the village (…) it was also about social contact as well for people’s mental health. Because yes, we couldn’t socialize but actually you were allowed to go out for food, so people could actually have a chat while wearing the masks, being safe, keeping themselves 2 m apart but actually for some people it was the only people that they saw in the week. So, it was really good for mental health as well. (Amelia, South East England).

Amelia particularly valued the *coping role* of the mutual aid group, arguing for the importance of these groups even for those who felt they did not need help with basic needs but needed the emotional support from the group. Participating in mutual aid groups was perceived as contributing to volunteers’ own sense of coping as it helped to give purpose and routine during the pandemic (cf. Mao et al., in press): “But when the volunteers were getting involved you could see that it’s given them that daily routine and something they could look forward to” (Emma, North West England). Furthermore, when referring to positive benefits, some participants also stressed benefits related to their own *development of personal skills*:

Interviewer: Have you learned something from coordinating this group?
Theo: Yeah, volunteer management, onboarding, learning a bit more about GDPR [General Data Protection Regulation]. Learning more about things like mental health issues. Community care, what resources are available in the neighborhood. (…) So, I didn’t really know the neighborhood. I can’t say that I know the neighborhood after a year and half, that’s impossible, I think. But I think I know it a lot better. (Theo, Greater London, England).

Among other things, some felt to have gained practical knowledge on community organizing, on how to deal with people in group settings, and how to assume leadership roles. They also improved their communication skills and learned how to listen to people’s needs. In this sense, participants perceived the group as a space for personal growth and learning, which in some cases led them to become more aware of their own role and impact within the local community: “I’ve learnt the positive impact you can have on your community if you’re willing to give up some of yourself to your community (Amelia, South East England).

The positive emotions and other psychological benefits were also related to a *strong sense of contribution* to the community. There was a strong sense of achievement explicitly stated by 22 participants: “so, you know, there’s some adjustments that we know need to be made. But it didn’t diminish the success of the thing at all.” (Arthur, South West England). The support provided to the community as well as specific events and activities organized by the group were perceived as successful. The sense of being able to contribute appears in different levels, and it was directly associated with positive emotions, as we can see in the following excerpt:

*And when you see that journey and you know that you’ve helped that person and you see that person change and be able to, be more proud of who they are, feel more connected, can’t access other services, it’s a massive, massive motivation. And I think the work that we do, even though we’re one of the poorest areas, we’re really, really blessed to see that difference that our work makes as well. And that gives you your passion, you’re driven when you know you’re making that difference, it comes naturally to want to continue to make that impact and have that positive impact on people and their families. (Emma, North West England).*

Emma’s comment clearly shows how interviewees perceived their group’s ability to effectively contribute to improve the lives of people within their community. While some participants focused on the ability of the group to mobilize help, several participants focused on the ability of the community as whole to come together when necessary.

Our analysis suggests that practices of solidarity involved in mutual aid groups were valued by all participants, who described their experience of participation positively. Positive emotions such as pride, joy and happiness were considered factors sustaining mutual aid groups over time. The shared sense that the group and the community itself responded effectively and promptly to community needs, as well as the perception that participating in mutual aid groups helped to provide well-being and new skills, also seem to be important factors for sustaining long-term participation.

### Theme 5: Increasing Sense of Local Community Belonging and Cohesion

Twenty-six participants described several positive effects on the *sense of community* resulting from participation in COVID-19 mutual aid groups. These participants tended to describe the local community as more connected and cohesive in relation to the pandemic, and that mutual aid groups experienced during the pandemic have shown that it is possible to “to bring everyone together” (Amelia, North East England). Other participants pointed to the positive benefits of finally getting to know their neighbors: “a lot of the neighbors actually know the people who are living beside them, whereas before we didn’t. That’s one of the positive things.” (Zoe, North West, England).

Several other participants expressed that their experience in COVID-19 mutual aid had helped to *build relationships* within the community and has increased the sense that they can rely on others within the community: “a lot of the neighbors actually know the people who are living beside them, whereas before we didn’t. That’s one of the positive things!” (Emma, North West, England). Likewise, “community spirit” was an expression used by some participants, who believe that the local community had become much friendlier after COVID-19, and there was an increase in the sense of community, despite the challenging situation: “I think genuinely the community spirit now, especially in [locality] is absolutely brilliant. And people have lost their jobs but they’re still willing to go out of the house and do something” (Mathew, West Midlands).

Importantly, interviewees not only perceived the community as more connected, they described their own relationship with the community as stronger. Many participants described how the COVID-19 situation has allowed them to build relationships with others in the local community:

Interviewer: And did you learn something from the community?
Rose: Oh yes. We have a wonderful community, they are generous to a fault, they are absolutely looking out for everybody else, they do worry about their neighbors. They worry about the people they can’t see on the street anymore, because nobody’s on the street. So, I have made some very good friends through this group (…) and I wanted that, I wanted to know people in my community, I wanted to feel like I could contribute something (…). So even though it took a terrible pandemic to do it, we now feel that people know that we’re reliable and that we can help out in an emergency. So, I’ve learnt that we chose well in picking this place to live. (Rose, Mid Wales).

As with Rose, many other participants mentioned feeling more connected to their own community as a result of their engagement with the mutual aid group. Moreover, for some groups these experiences within the community will have a future impact locally, and they talked about continuity and “legacy” to describe the experience:

*I expect we’ll continue to engage folks because I do think in terms of their values most of the volunteers really want to help people, they really wanted to, you know, make connections in their community, and this is a way for a lot of people to maybe not lived long to make new friends and maybe get involved in the life of the community, and I think that that will be a real legacy. You know the fact that, the kind of, it’s a really cheesy term but the kind of community-cohesion I think has really improved. (…). I don’t ever get a sense that we’re going to go back to the way exactly that things were, you know I think the volunteers and the people who have been involved in mutual aid led activities will be central to that [recovering] and we’re started to see that who have volunteered, you know, applying for jobs, maybe in things that they wouldn’t have done before. Or getting involved in projects and things like that as well, which is really interesting. (Logan, West Central, Scotland).*

This rich extract from Logan suggests how participation during the pandemic may have *strengthened the local community spirit and led to more engagement*. Importantly, other references to group continuity once the pandemic is over were evident in almost every interview. Views around the future of the group beyond the pandemic were very much related to the perceived needs of the community, with interviewees who perceived high and continued needs in the community feeling that they were prepared to meet community needs even when the pandemic is over:

Interviewer: And finally, how do you see the future of this group?
Sophie: Only positive to be honest. I mean we’re ready for round two. (…) I think this is going to be morphed into that, and they are going to continue this service, forever now. (…). This is no longer a COVID response, this a community response. So, if anybody at any point in next month, in a year or in 2 years, needs their shopping done, needs a prescription, needs taking to the doctors, the group will do it. Because we will now have the volunteers - this is the big thing that’s come out of this, because we found all these volunteers, they all want to continue to help, a lot want to continue to help, they’re able to sustain that, and continue to provide the service. (Sophie, East of England).

Sophie’s comments clearly expressed a commitment with the community beyond COVID-19 and suggested that her community has irreversibly changed. On this matter, interviewees’ comments suggested that even those participants who were not involved pre-pandemic expressed a desire to continue to be involved in the recently created group when the pandemic was over. Additionally, five participants from pre-existing groups also pointed to an effect on their own organizations, namely in terms of having more volunteers, and more knowledge on community needs and services. Five interviewees from pre-existing groups mentioned that COVID-19 has shown the importance of community organizing while others argued that their activity and services have grown since COVID-19 and they feel more prepared to respond to community needs. Other interviewees said they believed that COVID-19 had raised awareness of the group: “I think, I hate to say this, but I think that COVID-19 has raised the awareness of the group. So, I actually think that certainly in the next, certainly for the next year, so it will definitely flourish.” (Lisa, South East England).

In addition, while some mutual aid groups may have stopped providing help in the community, participants continued their own commitment to community action, by engaging in other groups and projects.

In summary, participants described an increased local sense of community and cohesion which were related to willingness to keep involved in the future. Participants’ accounts showed that COVID-19 mutual aid groups were perceived not only as an effective tool for addressing the COVID-19 crisis, but also as a way to increasing bonds within the community, which may lead to post-COVID participation and solidarity. In turn, for some participants, group continuity after the pandemic was an expected progression of creating COVID-19 community response in the first place. Participants’ accounts of group continuity suggested that there is a willingness to maintain support in the community in the future, either as temporary response for emergency situations or as permanent and continuous support for the community.

## Discussion

Drawing on 32 interviews with community organizers of COVID-19 mutual aid groups in the United Kingdom, the present study identified several strategies considered key for sustaining COVID-19 mutual aid groups, as well as practical and psychological experiences that were perceived by the organizers as important in motivating continued participation. Overall, our findings suggested that meeting community needs with localized action and resources and building trust and community-based alliances were foundational elements in COVID-19 mutual aid. According to the organizers participating in our study, group process strategies employed by mutual aid groups, which revolved around promoting a shared identity, effective communication between groups members, a culture of care and support within the group, group meetings and events, and an informal but organized leadership structure, helped to sustain involvement within their groups. The experiences resulting from participation in the group led to positive emotions, such as joy, and efficacy. Participation in COVID-19 mutual aid was also related to an increasing sense of local community belonging and cohesion.

Despite their diversity, all groups in our study were organized based on their local community, which seems to have facilitated access to human resources (e.g., volunteers) and practical resources (e.g., venues, vans, donations), that were then related to the endurance of mutual aid groups over time. As previous studies found in disaster communities (Ntontis et al., 2020; Tekin and Drury, 2020), alliances were a key strategy for ensuring the endurance of the groups over time. Besides, the collaborative and cooperative approach between people, groups, and organizations has created future opportunities for participation. The alliances built during the pandemic also facilitated the integration of other community initiatives and projects that were not necessarily COVID-19 related, which suggests the importance of community alliances in sustaining future participation.

Relating to the strategies employed by COVID-19 mutual aid groups, our findings suggest that groups focused on several strategies, at both group and community levels. At a community level, our analysis aligns with previous studies of COVID-19 mutual aid groups suggesting a high diversity in terms of activities realized, pre-existing nature, and the characteristics of the people involved in mutual support during the pandemic (e.g., Pleyers, 2020; Sitrin and Sembrar, 2020; Mao et al., 2021).

Our findings also align with previous research suggesting that group processes may be important for sustaining solidarity over time (Drury et al., 2019; Ntontis et al., 2020). We found that evoking a shared identification was a deliberate strategy used by several COVID-19 mutual aid groups. There was an overall commitment to increasing the sense of belonging among groups’ members, often through regular communication and feedback, shared meetings and events, clear rules, structure and guidelines, and a strong focus on the idea of caring for the well-being of group members.

Taking care of each other was considered to be vital for sustaining COVID-19 mutual aid groups. Participants described taking care of each other, making sure that the needs of all the members were taken into consideration, and that no volunteer was placed in a risky situation or was working too many hours for the group. Past research has shown that while long-term activists benefit from using personalized strategies of personal care to avoid burnout (Gorski, 2019; Driscoll, 2020), burnout should be approached from a group perspective, i.e., through a community-care burnout orientation (Gorski, 2019). Interestingly, our analysis suggests an orientation toward a group care approach, which may have the potential to help sustain long-term participation. Simultaneously, our findings also show that there is a perception of the need to recognize and celebrate the groups’ achievements, and that many groups were planning to do this when it is COVID-19 safe. Celebration events are vital for enduring participation over time (Ntontis et al., 2020), with these events expected to have a crucial role in sustaining community solidarity.

Moreover, participants in our study argued that the sustainability of mutual aid groups and the participation in these groups were also related to a set of practical and social psychological factors. In terms of practical factors, it is worth considering that mutual aid groups sustained themselves because they had access to resources needed to perform their tasks, namely in terms of people’s availability to participate and access to goods and funds. A local community-based approach facilitated the coordination and distribution of help, the mobilization of volunteers and the access to resources. There were, however, several concerns with the lack of stability in accessing key resources (e.g., vans, storage spaces, grants, skilled volunteers) after the pandemic. The ability to mobilize resources in the long term was a challenge faced by many groups, especially those working with marginalized groups in socio-economic deprived areas. Previous research has suggested that inequalities in the distribution or availability of social support can be explained by pre-existing social inequalities (Kaniasty and Norris, 1995), which might have affected who receives and who has access to support structures and resources. The decline in terms of resources and the saturation of supportive networks are also factors that have been found to influence the decline of disaster emergent groups (Norris and Kaniasty, 1996; Kaniasty and Norris, 2004).

Importantly, our findings suggest that the experience of participation in COVID-19 mutual aid groups was empowering in several ways. There was a general sense of being able to contribute and effectively respond to community needs during and even after the pandemic. Such perceptions were, in general, followed by descriptions of the power of mobilizing the communities and expressions of positive emotions associated with participation, such as joy and pride. In addition, our findings suggest that participating in COVID-19 community solidarity enhanced participants’ well-being and sense of being able to contribute to the local community, as previously found in other studies and contexts (e.g., Alfadhli et al., 2019; Bowe et al., 2020, 2021; Mao et al., in press). Boezeman and Ellemers (2007), for example, found that pride and respect for the organization were predictors of long-term volunteering. Additionally, a recent study from Bowe et al. (2020) showed that participating in volunteering is a source of pride, satisfaction, and well-being, and that volunteering predicts increased community identification and support, which in turn mediates the relationship between volunteering and well-being. Since one way of looking at sustained engagement is through the consequences of participation (Selvanathan and Jetten, 2020), it can be argued that COVID-19 mutual aid and community support have the potential to translate into long-term community responses. Positive emotional experiences, in particular, have the potential to shape people’s motivation to future engagement (Becker and Tausch, 2015), as ours and previous studies suggest (e.g., Bowe et al., 2020).

Finally, for those participating in organized help, new community bonds and ties have been created, which is in line with previous arguments that practices of solidarity often involve the construction of different and new social relations (Drury et al., 2019; Pleyers, 2020). We found that people involved in the COVID-19 mutual aid groups increased their sense of belonging by increasing the number of social connections and bonds with others in their local communities. Additionally, COVID-19 mutual aid groups acted as a platform for building such connections and a context facilitating the emergence of new community shared identities. Specifically, participants described an increase in terms of community identification and, simultaneously, increased identification with the cause and the goals of the COVID-19 mutual aid groups. Considering that sense of community has a positive and strong influence in diverse forms of participation (Talò et al., 2020), sense of community and cohesion are likely to be important factors for sustaining mutual aid groups over time.

### Limitations and Future Research

While we tried to reach diverse groups and participants, our sample still overrepresented groups located in England. Besides, thousands of mutual aid groups were created in the United Kingdom during the pandemic, and our study only captured the experiences of a small sample of these groups. It is also possible that there was a self-selection bias, leading more engaged groups and participants to respond to our call for participants. Additionally, several groups did not have their contact addresses available, and others were contacted but did not answer our invitation. It was particularly difficult to reach politicized groups and groups working in deprived and marginalized areas. The diversity of groups should be the focus of further research, as it is likely that the level of politicization may influence the future of mutual aid and their ability to sustain participation over time. In particular and considering that marginalized and deprived groups are those being most affected by COVID-19, it is crucial to look at how community groups and activists will respond to social inequities in the recovery and rebuilding processes.

Our choice to focus on organizers was appropriate and necessary to address the question of strategies used by active members to sustain the involvement of others. However, on the topic of the motivating experiences that arise from participation, while interviewees referred to other volunteers’ experiences as well as their own, this study’s insights on this aspect of the findings may be limited to the perspective of those highly active and engaged. Future research should interview or survey volunteers having different roles and types of participation (e.g., sporadic, continuous) to test whether the strategies and experiences analyzed here do indeed lead to sustained involvement.

It worth noting that at the time of the interviews, mutual aid groups had been active for several months. We started our interviews a few months after the easing of the first lockdown and the shielding requirements, and there were some signs that the activity of mutual aid groups declined after easing the first lockdown (Tiratelli, 2020). However, infection rates continued to increase and the need for self-isolation was high during the period of data collection, which suggests that the need for mutual aid and support amongst communities still existed. In this sense, the data collection period of our study is also beneficial as most participants had been involved in mutual aid for more than six months. In most cases, such participation has involved a high and continued level of commitment in a situation of crisis, which we believe is particularly relevant to understanding sustained participation. Our findings show that groups were able to implement strategies for sustaining participation over time, and that some groups were highly committed to providing organized help after the pandemic. It would be important to follow these groups over time, as well as interviewing people who dropped out, to examine how the strategies and factors identified in our study are related to long-term and sustained participation and solidarity.

## Conclusion and Recommendations

Previous research demonstrates that post disaster solidarity tends to decline over time (Kaniasty and Norris, 1993; Norris and Kaniasty, 1996; Kaniasty et al., 2019; Ntontis et al., 2020). A key strength of our study is that it extends previous literature by focusing on the strategies and factors that may sustain COVID-19 mutual aid groups over time. Our analysis shows that several community and group level strategies and experiences were related to sustained participation in COVID-19 mutual aid groups, including meeting community needs over time with localized action and resources, building trust and community-based alliances, employing group processes strategies, experiencing enjoyment and efficacy in collective coping, and increasing sense of local community of local community belonging and cohesion.

Based on these findings, some practical and important implications can be drawn. First of all, given the importance of resources in sustaining COVID-19 mutual aid groups (theme 1) there is a need to provide practical and financial support to COVID-19 mutual aid groups. However, this should be done without constraining or interfering in their actions, decisions or activities (Tiratelli and Kaye, 2020). It should also be combined with a broader strategy of supporting social infrastructures in critical areas such as education, housing, and transport (Power and Benton, 2021).

Second, COVID-19 has disproportionately affected ethnic minority communities (Hooper et al., 2020), and it is now urgent that we understand and take steps to mitigate the wider social and economic impacts within these communities, to best prepare to address the expected long term-social impacts of COVID-19 (Bedford et al., 2020). COVID-19 mutual aid groups have acquired extensive knowledge on the local needs, resources and potentialities of their communities (theme 2). This knowledge should be mobilized for developing programs and interventions for addressing the medium and long-term impacts of COVID-19.

Third, following a local community-based approach facilitated the coordination and distribution of help, the mobilization of volunteers and resources, leading to a sense of being part and able to contribute to the local community (themes 1, 4, and 5). Governments should prioritize community-level interventions, as they have the potential to benefit individuals and communities.

Finally, our study supports previous suggestions for the need to recognize the role of group processes (Drury et al., 2019; Ntontis et al., 2020). There are several strategies that can be employed by mutual aid groups and that have the potential to sustain participation over time. For example, community organizers, activists and coordinators can actively invoke shared identities, promote a culture of taking care of each other, organize socializing meetings, and facilitate open communication between group members (theme 3). As our study shows, these group strategies may have the potential to lead to long-term community responses, which will be key for ensuring that communities effectively recover from the COVID-19 pandemic.

## Data Availability Statement

The datasets presented in this study can be found in online repositories. The names of the repository/repositories and accession number(s) can be found below: https://figshare.com/s/25c1521f547a84e00f7c.

## Ethics Statement

The studies involving human participants were reviewed and approved by University of Sussex Cross-Schools Research Ethics Committee (reference number ER/HAFC1/8). The participants provided their written informed consent to participate in this study.

## Author Contributions

JD, EN, MF-J, and GM contributed to the conception and design of the study. MF-J and GM conducted the data collection. MF-J analyzed the material. JD, EN, GM, CC, MM, AS, and JS revised and discussed the first draft of the analysis. MF-J wrote the first draft of the manuscript. All authors contributed to the interpretation of data, reviewed, wrote and approved the final version of the manuscript.

## Conflict of Interest

The authors declare that the research was conducted in the absence of any commercial or financial relationships that could be construed as a potential conflict of interest.

## Publisher’s Note

All claims expressed in this article are solely those of the authors and do not necessarily represent those of their affiliated organizations, or those of the publisher, the editors and the reviewers. Any product that may be evaluated in this article, or claim that may be made by its manufacturer, is not guaranteed or endorsed by the publisher.

## Footnotes

1 Three dots within parentheses are used when a part of the quote was omitted because it was not relevant to the passage.

2 Square brackets enclose words intended to clarify meaning and provide a brief explanation.
